# Round the Hospitals

**Published:** 1920-01-03

**Authors:** 


### ROUND THE HOSPITALS.

The Royal Assent was given on December 23 to the three Nurses' Registration Acts, that for England and Wales, that for Scotland, and that for Ireland. Thus ends a long and troubled chapter of nursing history, and thus begins a new chapter which at present is a blank page. The immediate need is for understanding of what registration can and what it cannot perform for the nurses. To regard it as a kind of Aladdin's lamp, which by a mere touch can realise every wish, is to lay up a

**ROUND THE HOSPITALS—(*continued*).**

store of disappointed hopes. To sentence it from the beginning to failure merely because in other lands its full capabilities have not yet been discovered and utilised is equally futile, and has even less excuse. Rightly regarded the new Registration Acts are powerful engines, and, like other engines, the success of the work they perform must depend on the minds which direct them.

Nursing sisters entitled to the Victory Medal Riband will be delighted to hear that a preliminary issue will now be made officially. *Personnel* still serving will receive the riband from commanding officers and heads of departments. Demobilised members of the nursing services should make individual application as follows: Members of the Q.A.I.M.N.S., to the Secretary, War Office (A.M.D. 4), Cornwall House, Stamford Street, E.C. 1, giving particulars of service; Territorial Force Nursing Sisters, to the Secretary of the War Office (T.V. 4), 80 Pall Mall, S.W. 1.

Christmas festivities began at the Glasgow Western Infirmary on December 23, when the annual prize-giving in connection with the Nurses' Training-School took place and a large and distinguished company attended. Lord Glenarthur, Chairman of the Board of Managers, Lady Glenarthur, Lord Provost Stewart, and Mrs. Stewart, Sir Samuel Chisholm, Lady Dunlop, Sir Hector Cameron, Sir Kennedy and Lady Dalziel, were among those present, with Colonel D. J. Mackintosh, C.B., M.V.O., Medical Superintendent, and Miss Gregory Smith, Matron. Lord Glenarthur, in expressing the satisfaction all felt at the return of the doctors and nursing sisters who had been away on war service, paid a warm tribute to the unselfish and patriotic devotion of those members of the staff who had borne the strain at home during the war, and particularly expressed his admiration at the way in which Colonel Mackintosh and Miss Gregory Smith had shouldered their responsibilities. They had every reason to be proud of their staff. A fifty-six-hour week had been secured for the nurses, but further progress was barred by the need for an addition to the Nurses' Home. The Countess of Glasgow presented prizes to Nurse Muriel Annette Walker, Nurse Lillias Barrie Wiseman, and Nurse Florence Ethel Duncan, of whom the latter carried off three. It was announced that a grant of £10,000 had been made by the Scottish Red Cross to provide and equip the building for massage, medical electricity, Swedish remedial exercises, and that £5,000 had been given to the University to establish a lectureship in Electrical Diagnosis and Therapenties in connection with the infirmary.

Dr. Addison has stated, in reply to a question in the House of Commons, that he is by no means satisfied with the results obtained in combating tuberculosis under the National Health Insurance Act. The amount spent by public authorities in England and Wales on the treatment of tuberculosis in the year 1917-18 was approximately one and a half million pounds, and it is estimated that the cost of treating this disease has by now risen to two millions. There is insufficient sanatorium accommodation, and some difficulty in securing proper treatment at a sufficiently early stage. Everything depends on a correct diagnosis being made at the beginning, and on inducing the patient to submit to early treatment. It is precisely in these directions that district nurses, when adequately trained, prove the best auxiliaries of the Health Minister. It is not their place, of course, to attempt diagnosis, but it is on them that the onus rests of detecting suspicious cases and inducing them or their parents to seek medical advice. It is to their recommendations, when they have once acquired the confidence of their patients, that patients will lend an ear, and they are thus able to facilitate enormously the labours of all who are combating tuberculosis. Post-graduate instruction to district and village nurses in the early manifestations of this disease is greatly needed in the Provinces.

Many persons engaged in philanthropic work had cause to lament their lack of foresight at the beginning of the war in regard to the rising prices of materials. The Ladies' Guild of St. Thomas's, which has just issued its report for 1918-19, was very fortunate in having an honorary work secretary, Miss Clerk, who laid in a great store of materials in anticipation of the time when they would be almost unprocurable. Great as are the difficulties of the Guild in having to pay, for instance, at the present time 2s. 3d. a yard for flannelette inferior to what they used to get for 6d. a yard, the difficulties of the poorer part of the population served by St. Thomas's Hospital are greater by far. The Guild has ever-increasing demands made on it, and urgently appeals for help in money and labour. The Hon. Treasurer is Miss South, 1 Wellesley House, Sloane Square, S.W. 1.

The Lambeth Guardians have resolved on a very striking experiment in the interests of their infirmary nurses. As long ago as last July they adopted a resolution for a forty-eight hour working week at this institution, and were faced with the necessity for engaging sixty-three additional probationers before it could be inaugurated. Where were these nurses to be housed? There being no answer to this question Mr. Frank Briant, M.P., chairman of the Lambeth Board of Guardians, conceived the notion of authorising the matron to engage non-resident probationers living at their own homes within easy distance of the infirmary. When fully worked out the scheme will provide for lectures being given in the morning and evening and a canteen will provide the extra probationers with meals. Mr. Briant is quite ready for adverse criticism, and is content to let the event decide whether the experiment be workable. He recalls with pride that it is not the first time that his board has made

**ROUND THE HOSPITALS—(*continued*).**

experiments which have received the seal of approval conferred by widespread imitation. And he warns his critics that the difficulties of getting good nurses are increasing.

LAMBETH Infirmary is particularly well placed for inviting the services of non-resident probationers. A large middle-class population numbering hundreds of the families from among which probationers are normally drawn lies within convenient reach. It is most desirable that the experiment should be tried, for the future of nursing in the next few years depends largely on the housing question. To shorten the working hours of the nurses is one of the most pressing needs of the service. Yet to find residential accommodation instanter for sixty-three extra nurses, as at Lambeth, is plainly impossible. If it can be demonstrated that probationers can discharge all their normal duties and go through a normal course of nursing studies, while living for part of their course at home, a way will have been found out of an impasse which is gravely retarding reform. This solution of the difficulty is of course only open to hospitals favourably situated in or near a residential quarter. It can never, we judge, be more than an emergency expedient or cover more than part of the nurses' years in training.

THE avalanche which fell at Davos on the afternoon of December 23 was horrifying in its sudden envelopment of a district largely occupied by invalids. *The Times* correspondent states that "Although snow had fallen thickly during the night no one anticipated danger until the alarm was sounded from the church tower. One avalanche crashed into the lake and did little damage. The second fell with full force upon the sanatorium, and the Pension Germanica. The window-frames and shutters of both buildings were pushed in, and rooms on the mountain-side were filled up high with snow. The whole sanatorium rocked, and a state of terror prevailed. People in the basement and on the ground floors were the most exposed to danger. One patient only was killed. The rest were safely removed to another sanatorium outside the track of the avalanches which continued to fall. We learn with regret that the matron of the Dorf Sanatorium, Sister Bertha Silberer of Zurich, was among those killed.

GREAT efforts are being made by the London Labour Party to secure a forty-eight-hour week for mental nurses. The forty-eight-hour Bill would, if passed in its present form, include mental nurses in its operations, but it is stated that the Mental Hospitals Association is endeavouring to get them excluded from its provisions. Mr. Herbert Morrison, Secretary of the London Labour Party, has written to Sir Robert Horne to announce the intention of his executive committee to resist the demand of the Association on the ground that the work of mental nurses of both sexes entails much nervous strain, and that the case for restriction of hours is particularly strong in respect of these workers. It is astonishing that any who have intimate knowledge of the circumstances under which asylum work is conducted should be disposed to resist the claims of the men and women in charge of the patients to special consideration. The whole future of mental nursing, which may be said to rest on the power of attracting to the service the right kind of nurse and attendant, depends on reduction of hours.

*The Times* annual review of the Poor-Law which appeared on December 27 sounds a transition note. The sands of the guardian's office are fast running out and we are on the eve of an immense administrative change which will once and for all banish the expression "Poor-Law Nurses." Dr. Addison has already forecasted an alteration of the first scheme for the reform of the Poor-Law, under which the aged, the infirm, and the sick were to have been dealt with separately by the new Poor-Law authorities. It is now recognised that the care and treatment of the sick and infirm ought to be absorbed in the general health services of the country. The children will be dealt with by the education authorities, and in future all who are in need of medical assistance are to be entitled to receive treatment before instead of after sickness has reduced them to destitution. This is the ideal, and hard as it may prove to carry it into effect the introduction of a sound ideal cannot but react most favourably on the status and ideals of the nurses charged with the duty of helping to make it a reality.

THE mild weather was a great comfort in lessening the fatigues of Christmas festivities in hospital, which are apt to begin at an unconscionably early hour. In some institutions carols started well before daybreak, and in most a murmur of satisfaction at the contents of stockings or parcels hung on to the beds became audible at a time when sleepers are usually cherishing their last moments of oblivion. In some of the London hospitals noted Christmas entertainers among the medical staff were back for the first time since war began, and had many new devices for stirring merriment. So exhilarating and healing is the effect of laughter on the sick that it is much to be regretted that the medical profession have not yet discovered some means of prescribing it not only at Christmas. Its effect on nurses is to obliterate the fatigues of a sixteen-hour day by dreamless repose. Surely there is a moral in this.

DUBLIN is celebrating New Year's Eve with a grand fancy dress ball in the Gresham Hotel, in aid of the Nation's Tribute to Nurses. Various ladies, including Viscountess Gormanston, Lady Castlemaine and Lady Decies, are making up parties for the occasion which even before the event had reached a position of assured success.

THE advertisements for emergency nurses inserted in our columns by the Central Council for District Nursing in London have done their work well. So large a number of applications have been received

**ROUND THE HOSPITALS**—(*continued*).

that already the names of twenty-six nurses have been entered on the panel. These have all been interviewed by Miss Amy Hughes and their references have been taken up. It is very important that district nursing associations in London should realise the admirable organisation which stands ready to supplement their resources. Up till now district nurses who "went sick," or took emergency leave, were always conscious that they exposed their particular district to inconvenience and their colleagues in all probability to over-work. This need no longer be the case. In the event of any shortage of staff, whether for a short or long period, the secretary of any district nursing organisation within the County of London can obtain a relief nurse at once by applying to the assistant secretary of the Council, Miss Pollock, c/o The City Parochial Foundation, 3 Temple Gardens, E.C.4. We wish there were any hope that the County Nursing Associations could succeed in building up reserves in the same manner.

The device adopted in certain new buildings where ground is cramped for saving bath-room space, is likely to be found particularly suitable to nurses' homes, where the preposterous demand for a bathroom for every six nurses is beginning to reduce architects and managers to despair. The new bath is of the nature of a wide foot-pan set in a cubicle, and the bather, standing upright after a copious lather, turns on the hose, with or without spray attached, and receives a stream of water on every part of the body. Some four feet square suffices for each bath cubicle, and as the process of bathing takes no more than five minutes under this plan, the difficulty of finding time for successive baths vanishes. Bathing under the hose is stimulating and pleasant. Moreover, it is entirely free from the danger of exhaustion produced by recumbent baths in deep hot water. It need hardly be said that those who accustom themselves to the use of the cold spray find it specially invigorating.

Influenza so far this winter has appeared in a mild form. Wherever it shows itself the nursing association should take precautions in advance, first, by securing if possible the promise of a relief nurse in emergency, and, secondly, by organising a band of voluntary home helps. The fear of infection made great strides last winter. Whereas before the epidemic neighbours were always ready with kind offices, now, it may be noticed, they stand within the door and carry out only such services as can be done from aloof. To keep the fires stoked, supply suitable cooked foods, milk, and the indispensable cup of tea at intervals, some organisation is required. As likely as not the nurse may herself be one of the victims and will need attention. To enable all who are attacked to stay quiet in bed by keeping things going to the extent we have suggested would check many cases while yet in a mild stage and probably save some lives. But members of nursing committees must lead the way themselves.

At Stretford the war memorial decided on is the establishment of a nursing service for the district on broad and comprehensive lines, so that every resident in the district may be enabled to secure the services of a nurse in the event of illness, the well-to-do on payment of a fee proportioned to the cost, and the poor, either without fee or a nominal charge. It is estimated that from £20,000 to £30,000 will be required to place the scheme on a good basis as well as to erect a granite obelisk with the Roll of Honour inscribed on it. The Stretford division of B.R.C.S. has promised £6,000. About £1,500 has already been contributed by members of the nursing services to establish a memorial to the nursing sisters of Q.A.I.M.N.S. and the T.F.N.S., who gave their lives for their country. The Fund is being organised from the headquarters of the matron-in-chief, Q.A.M.N.S., Cornwall House, Stamford Street, S.E. 1. It is believed many of the demobilised sisters may yet be ignorant of its existence, as they have scattered far and wide since they left the service. No decision has yet been reached as to the form of the memorial, which must to some extent depend on the amount ultimately subscribed.

The Tynemouth Guardians have been informed by the Medical Department of the Board of Education that they can claim the grant of £20 payable under the recent regulations in respect of their midwifery pupils, as well as a further grant of £6 for each such pupil who serves beyond the limit of six months. These grants are only to be made for candidates who give an undertaking that they mean to practise midwifery. For the future staff nurses at the Tynemouth Infirmary appointed under the arrangements for midwifery training will be required to sign an agreement to stay not less than 12 months. Should they declare their intention of practising midwifery, they will, if recommended by the medical officer, receive the sum of £5 out of the Government grant. The need for more practising midwives is very pressing, and it appears likely that the Government grants may stimulate women to adopt this career. The reproach that it affords only a starvation wage to those who embrace it is rapidly being wiped out.

At Kingston-on-Thames the Guardians have approved the recommendation of Dr. Davies, Medical Superintendent of the Infirmary, that the working hours of the nurses in this institution shall be decreased. At present the day-nurses work seventy-seven hours in the week and the night-nurses eighty and a-half. Dr. Davies asks for ten more nurses, which will enable the duty hours per week to be reduced to fifty and a-half for day-nurses and sixty-nine for night-nurses. The day-nurses will enjoy one day's rest in seven, a half-day on Sunday, and two hours off duty on other days. Night-nurses are to have one night off duty each week. The whole plan has been skilfully drawn up, and secures a great reform which will, no doubt, benefit patients as well as nurses. The new matron's salary is to be £100, rising by £10 increments to £140.

